# Percutaneous transverse pinning for metacarpal fractures: a clinical trial

**DOI:** 10.1007/s00402-024-05422-2

**Published:** 2024-07-04

**Authors:** Andrea Pintore, Alberto Astone, Gianluca Vecchio, Giovanni Asparago, Giampiero Calabrò, Filippo Migliorini, Nicola Maffulli

**Affiliations:** 1 Department of Medicine, Surgery and Dentistry, University of Salerno, Via S. Allende, 84081 Baronissi, SA Italy; 2 Department of Orthopaedics and Traumatology, San Francesco D’Assisi Hospital, Oliveto Citra, Italy; 3 Department of Orthopaedic and Trauma Surgery, Academic Hospital of Bolzano (SABES-ASDAA), 39100 Bolzano, Italy; 4 Department of Life Sciences, Health, and Health Professions, Link Campus University, 00165 Rome, Italy; 5 School of Pharmacy and Bioengineering, Faculty of Medicine, Keele University, Thornburrow Drive, Stoke On Trent, England UK; 6 Barts and the London School of Medicine and Dentistry, Centre for Sports and Exercise Medicine, Queen Mary University of London, Mile End Hospital, 275 Bancroft Road, London, England E1 4DG UK; 7 Department of Trauma and Orthopaedic Surgery, Faculty of Medicine and Psychology, University La Sapienza, Rome, Italy

**Keywords:** Metacarpal, Fracture, Transverse, Pinning, Percutaneous

## Abstract

**Introduction:**

Metacarpal fractures account for 25%-50% of all hand fractures and may negatively impact hand function and ability to work. Percutaneous transverse pinning of non-articular metacarpal fractures allows mobilisation immediately after the procedure.

**Methods:**

Between March 2017 and February 2022, 56 patients undergoing percutaneous transverse pinning for unstable metacarpal fractures were prospectively recruited. We investigated surgical outcomes in terms of Patient-rated Wrist/Hand Evaluation (PRWHE) and pre-and post-operative radiographic evaluation. The Student t-test was used to compare the means of PRWHE values after surgery. Statistical significance was set at p < 0.05.

**Results:**

The mean age was 40.21 ± 17.9 years (range of 16 to 86 years). The average operating time was 27.96 min. The mean follow-up period was 14.3 ± 6.4 months (from 2 to 41 months). The mean PRWHE score was 6.5 ± 1.8. None of the patients had clinically observable rotational deformities, and the functional outcomes were satisfactory.

**Conclusion:**

Percutaneous transverse pinning for non-articular metacarpal fractures restores excellent function, and imaging results are satisfactory. Further high-quality clinical trials are required to validate these results on a larger scale.

**Level of evidence:**

II, prospective cohort study.

## Introduction

Metacarpal fractures account for 25–50% of all hand fractures and negatively impact hand function and ability to work [1, 2]. These fractures rarely result in non-unions [3] but are associated with malalignment: varus, valgus, procurvatum, recurvatum, and rotational deformity [4, 5]. Rotational malalignment should be carefully avoided, as it interferes with power gripping [6]. Approximately 85% of metacarpal fractures can be managed without surgery [7]. However, prolonged immobilization may result in stiffness, pressure sores, and compartment syndrome [8]. Ideally treatment should allow early active mobilisation [9]. Surgery is indicated when the fracture is otherwise non-reducible, in instances of polytrauma or open fracture, in cases of shortening with extension lag or rotation, and with concomitant injury to nerves, vessels, and soft tissue [10, 11]. Various treatment methods have been proposed for metacarpal fractures, including the use of interfragmentary compression screws, plates, external fixators, and Kirschner wires (K-wires) [12–15]. K-wires are still widely used; they are usually introduced percutaneously and can be used in a variety of ways: cross K-wire, intramedullary, transverse, longitudinal (anterograde or retrograde), or combined with a cerclage wiring [16–20]. Previous studies, which described percutaneous transverse pinning using two K-wires distally and one proximally for treatment of closed displaced fractures of the neck of the fifth metacarpal bone (boxer’s fracture), showed excellent functional and anatomic outcomes [21, 22].

In our institution, we undertake percutaneous transverse pinning in all non-articular metacarpal fractures of the head, neck and shaft. The technique involves the reduction of the fracture under fluoroscopy and fixation with 2–3 differently distributed K-wires, proximal and distal to the fracture. The wires are advanced to the far cortex of the metacarpal adjacent to the one to be treated. The patients can mobilise the fingers and hand immediately after the procedure. The surgical procedure is easy to master, and the results are cosmetically and functionally satisfactory. We investigated the surgical outcomes in terms of Patient Rated Wrist/Hand Evaluation (PRWHE) and pre-and post-operative radiographic evaluation.s We hypothesised that percutaneous transverse pinning provided excellent results at short-term follow-up.

## Materials and methods

### Study design

Between March 2017 and February 2022, 56 patients undergoing percutaneous transverse pinning for unstable metacarpal fractures were prospectively recruited. All procedures were performed by two fully trained surgeons (AA and GC), as day-cases, with close reduction under image intensification, and transverse percutaneous osteosynthesis with K-wires. Patients without rotational displacement or palmar angulation of the fracture less than 30° were excluded. All patients received a pre- and post-operative evaluation both clinically and radiographically. Three radiographic views were taken: anteroposterior, oblique and lateral hand views. The PRWHE is scored so that the pain and function items are weighted equally [23]. This is achieved by dividing the sum score for the function by two before adding it to the sum of the pain score. The total PRWHE score grades from 0 (no pain/disability) to 100 (greatest pain/disability). The PRWHE is reliable and valid [24] and is easy and quick for the patient to perform [25, 26]. Before surgery, all patients included in the study signed an informed consent form that explained the operative procedure, functional and cosmetic expectations, and possible complications related to the surgery, consenting also to be part of any outcome research. The procedures were performed as day surgery with peripheral anaesthesia (peripheral nerve blocks or brachial plexus anaesthesia), which allowed patients to be discharged within 24 h. At the last follow-up, three orthopaedic surgeons (AP; GV; GA) evaluated all 56 patients to assess possible limitations in daily activities, degree of pain, return to work, changes in grip strength, satisfaction with the type of treatment received, and whether any cosmetic dissatisfaction was present.

### Surgical procedure

**Fig. 1:**
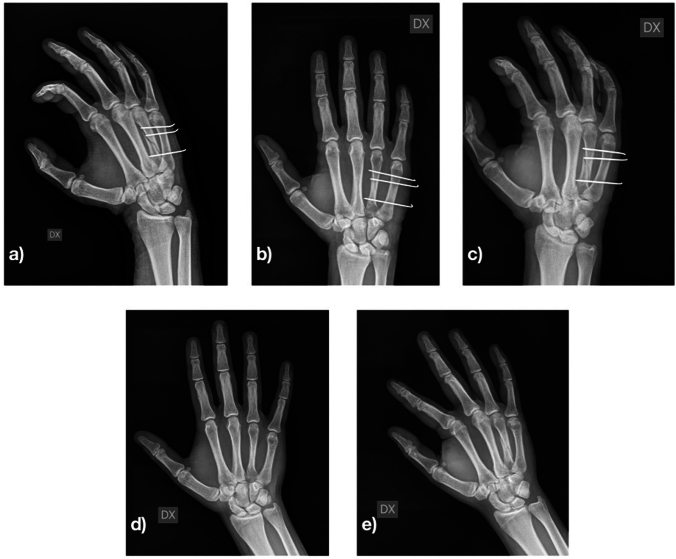
Fourth metacarpal spiral fracture. **a** post-operative radiography; **b** and **c** 1-month post-operative radiography; **d** and **e** 3-month post-operative radiography

**Fig. 2:**
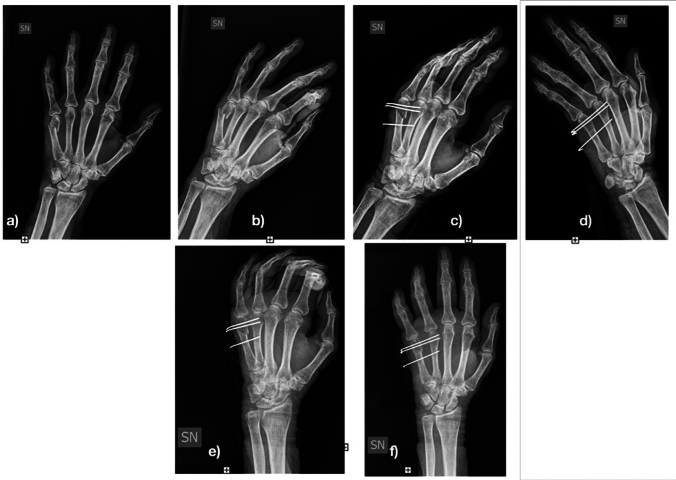
Fourth metacarpal spiral fracture. **a** post-operative radiography; **b** and **c** 1-month post-operative radiography; **d** and **e** 3-month post-operative radiography

All patients underwent loco-regional anaesthesia. As antibiotic prophylaxis, 2 g of intravenous cefazolin were administered before surgery. Closed reduction of the fracture was performed under image intensification. In some fractures, especially spiral or severely displaced (11 of 56), open reduction was necessary, performing a dorsal mini-incision to reduce the fracture as anatomically as possible. The most important target was to correct the rotational deformity. Fixation was performed with 1 or 2 K-wires 1.2 mm in diameter proximal to the fracture and with 1 or 2 K-wires distal the fracture to avoid displacement in the sagittal plane of the distal portion of bone. The K-wires were advanced through the fractured metacarpal up to the far cortex of the adjacent metacarpal (Figs. 1 and 2).

**Fig. 3:**
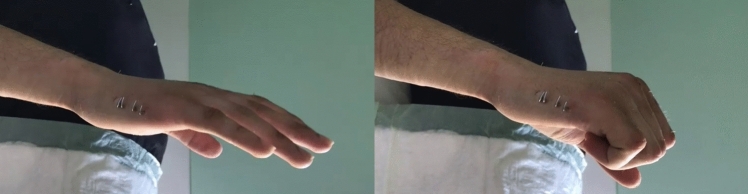
Fifth metacarpal fracture. Clinical evaluation of MCP joint mobility after insertion of K-wires in extension and flexion

Final imaging and clinical examinations were performed to check the rotation of the finger in an extended and semi-flexed position; then, the K-wires were cut and bent on the skin (Fig. 3). Patients were given a soft bandage to protect the K-wires and soft tissues for two to four weeks, with the metacarpophalangeal joints free. Patients were encouraged to mobilise the fingers in the immediate postoperative period.

### Postoperative care

Patients were discharged on the same day of surgery. The first clinical follow-up was performed after one to two weeks to evaluate the patient’s mobility and to inspect the K-wire entry wounds. A second clinical and radiographic examination was performed 30–35 days after surgery to assess the state of consolidation of the fracture and to program the removal of the K-wires, which took place either on the same day or six weeks after surgery. All patients were allowed to use the injured hand during this period, avoiding weightlifting and forced grips. After the removal of the K-wires, physiotherapy was recommended.

### Statistical analysis

The Student t-test was used to compare the means of PRWHE values after surgery. Statistical significance was set at p < 0.05.

## Results

**Fig. 4:**
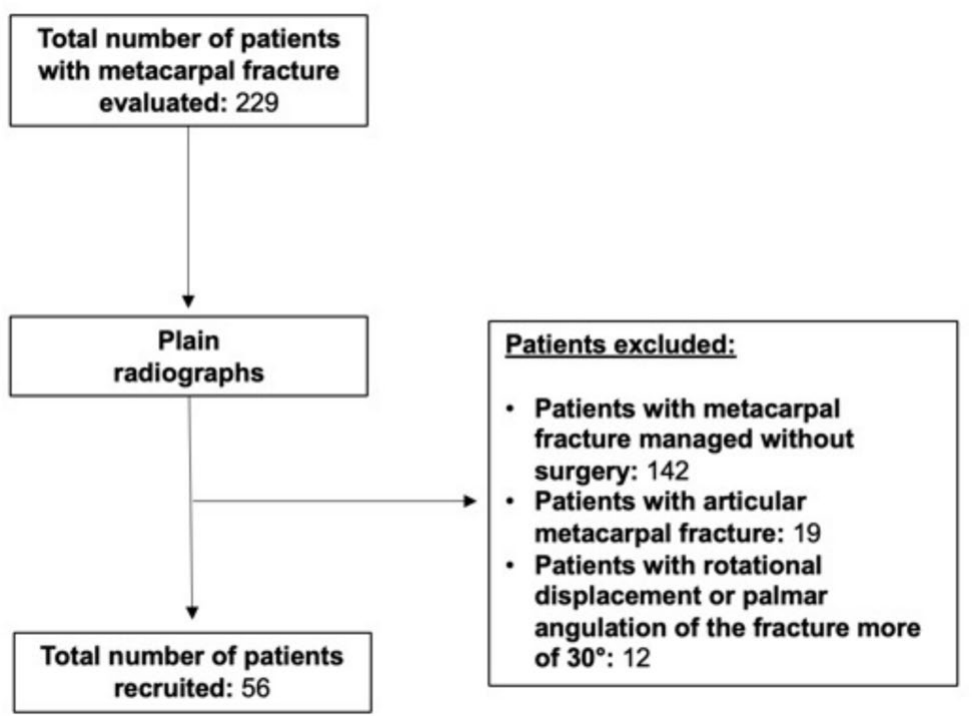
Enrollment process

Between March 2017 and February 2022, 56 patients, 48 males (85.7%) and eight females (14.3%), underwent percutaneous transverse pinning for unstable metacarpal fractures and were included in this study (Fig. 4). The mean age was 40.21 ± 17.9 years (range of 16 to 86 years) (Table 1).

**Table 1:** Patient demographics

Endpoint	
Patients (n)	56
Mean age (years)	40.21 ± 17.9
Female (%)	9 (14.3)
Dominant hand (%)	64
Mean follow-up (months)	14.3 ± 6.4
Average operating time (minutes)	27.96
Traumatic mechanism (n)	
Direct trauma	28
Fall	22
Other	6
Fractured metacarpal (n)	
V°	44
IV°	6
III°	6
Fracture pattern (%)	
Oblique	40
Transverse	32
Spiroid or comminute	28
Anatomic site (%)	
Head	21
Neck	34
Shaft	45
PRWHE score (mean)	
1 month	24.3 ± 5.4 (p < 0.05)
3 months	16.1 ± 2.7 (p < 0.05)
12 months	6.5 ± 1.8 (p < 0.05)
Satisfaction of patients (%)	96

Patient rated wrist/hand evaluation: PRWHE; p-value: p

In all patients, a single metacarpal was involved. The dominant hand was affected in 64% of patients (N = 36). The fracture pattern was oblique (short or long) in 40% of the patients and transverse in 32%; the remaining 28% were spiral or comminuted fractures. The anatomical site of the fractures was the non-articular head in 12 patients (21%), the neck in 19 patients (34%) and the shaft in 25 patients (45%). The average operating time was 27.96 min. The mean follow-up period was 14.3 ± 6.4 months (from 2 to 41 months). The PRWHE score was collected at one month, three months and 12 months after surgery. The mean PRWHE score was 6.5 ± 1.8 at the last follow-up. The overall decrease during follow-up in PRWHE score was statistically significant (p < 0.05).

At the final follow-up, 54 patients had a mean extension of 2° (range 4–0°) of the metacarpophalangeal joints, and 90° of flexion. All patients had full flexion of the interphalangeal joints. None of the patients had clinically observable rotational deformities, and the functional outcomes were satisfactory. Grip strength remained unchanged in 88% of the patients (49 out of 56), and all of them returned to work or their normal daily activities within two months after surgery. 96% of the patients showed a high degree of satisfaction with the surgical technique and the post-operative treatment received.

Two patients showed limitations in extension and flexion of the metacarpophalangeal with pain. In both patients, the fractures were clinically and radiographically healed, and the patients were encouraged to continue with physiotherapy. One patient presented a superficial infection at the K-wire insertion site, which improved with oral antibiotic therapy and removal of the K-wire at 28 days. A non-traumatic fracture of a second metacarpal occurred 4–5 days after K-wire removal in a patient operated with percutaneous transverse pinning for a fracture of the third metacarpal. The new fracture was transverse, in the location where one of the k-wires from the index surgery passed through bone, and was treated by another traverse pinning. No other complications were observed during the follow-up.

## Discussion

The present study reports the results of percutaneous transverse pinning for non-articular metacarpal fractures. The functional outcomes of all patients were excellent. Fractures of the neck of the 5th metacarpal (boxer’s fracture) are the most frequent [27], but long oblique and irreducible transverse fractures are the most unstable [28, 29]. Operative management for metacarpal fractures is indicated depending on the degree of volar angulation, shortening and displacement [30]. Excessive volar angulation can result in dorsal deformity with a prominent palmar metacarpal head and decreased grip strength [31, 32]. The degrees of volar angulation for each digit that may be considered for surgical fixation are 15° for the second metacarpal, 25° for the third metacarpal, 35° for the fourth metacarpal, and 45° for the fifth metacarpal [30]. An angulation defect greater than 30° for the fifth metacarpal is associated with a decrease in grip strength and range of motion [33].

Several surgical options exist for fixating metacarpal fractures: K-wire percutaneous fixation, (locking) plate fixation, and intramedullary fixation with headless compression screws. No consensus currently exists on the optimal method for metacarpal fracture fixation [34]. Different K-wires pinning treatments have been described: cross pinning, crucifix retrograde or antegrade pinning (Kapandji technique), and bouquet pinning with one or more antegrade K-wires [35–37]. Kapandji used an intramedullary axial K-wire with a curved tip to maintain the proximal fragment and reduce its angulation in flexion, and a transverse K-wire, perpendicular to the previous K-wire, through the head of the fifth metacarpal and into the head of the fourth metacarpal to reduce malrotation [38]. The K-wire technique is minimally invasive and is associated with a good post-operative range of motion [39], but it shows drawbacks such as pin-site infection, nonunion/malunion, the need to protect exposed pins, and prolonged immobilisation [8, 40]. The technique we used does not include an intramedullary longitudinal K-wire, which increases the risk of damage to the metacarpophalangeal joint and extensor apparatus and necessitates larger-diameter K-wires.

Miniplate fixation can be used when significant comminution precludes closed reduction and percutaneous pinning [30], and allows the most biomechanically stable construct [41], but may be associated with stiffness, avascular necrosis of the metacarpal head, extensor tendon injury, nonunion/malunion, and hardware irritation [6, 42]. Intramedullary fixation antegrade or retrograde is an alternative minimally invasive technique for the treatment of unstable extra-articular fractures of the metacarpals and allows early active mobilisation [12, 43]. Both K-wire fixation and miniplate fixation are equally effective in terms of total active motion and range of motion when used in closed metacarpal and phalangeal fractures [44–46]. Nonetheless, functional impairment requiring reoperation was reported in ORIF-treated patients [47]. Furthermore, ORIF takes longer and requires longer hospital stays [48]. There is no consensus among hand surgeons about the single most effective technique, though the evidence supports the use of percutaneous pins over ORIF with plates and screws in the management of such metacarpal fractures [49, 50].

Our patients experienced excellent clinical results and a very low complication rate. This is encouraging, even considering the lack of other studies on this surgical technique. Transverse pinning was first described by Lamb in 1973, who reported 39 diaphyseal fractures treated in this fashion, with no non-unions or infection [51]. We report one superficial infection at the K-wire insertion site. Pin-site infection is less common when the K-wires are buried beneath the skin (17.6% of exposed K-wire infection cases vs 8.7% of buried K wire cases) [52], but not all patients may tolerate this, and removal can be less straight forward.

This study has some limitations. First, there was no comparison with other techniques, such as miniplate fixation or intramedullary fixation. Only two studies described percutaneous transverse pinning limited to closed boxer fractures, with good clinical and anatomic outcomes. Another important limitation of this study was the short-term follow-up. We are aware that the highest level of evidence for the effectiveness of our or the other surgical treatment, such as cross pinning, crucifix pinning, and bouquet pinning, can only be produced employing a randomised study trial design. However, despite the constraints of our setting, we are confident that the results are valid and reliable. The recruitment process was rigorous, data collection was performed in a strict scientific fashion, we used validated outcome measures, and the results obtained are clinically relevant. Further high-quality clinical trials are required to validate these results on a larger scale.

## Conclusions

Percutaneous transverse pinning for non-articular metacarpal fractures showed excellent functional and anatomic outcomes and can be considered satisfactory. Further high-quality clinical trials are required to validate these results on a larger scale.

## Data Availability

All data are available under reasonable request to Dr Andrea Pintore (apintore@unisa.it).

## Abbreviations

K-wires: Kirschner wires
PRWHE: Patient Rated Wrist/Hand Evaluation
